# Should LifeVest Be Worn During Shower: A Case Report

**DOI:** 10.7759/cureus.88663

**Published:** 2025-07-24

**Authors:** Vinh Sieu Lam, Kim Huyen Huynh, Phillip Tran

**Affiliations:** 1 Cardiovascular Research Department, Methodist Hospitals, Merrillville, USA; 2 Faculty of Medicine, Moscow State University of Medicine and Dentistry, Moscow, RUS; 3 Medicine Department, Pham Ngoc Thach University of Medicine, Ho Chi Minh, VNM; 4 Cardiology Department, Nam Can Tho University, Can Tho, VNM

**Keywords:** coronary artery disease, icd, ischemic cardiomyopathy, lifevest, permanent atrial fibrillation, sudden cardiac arrest, ventricular fibrillation, wearable cardioverter-defibrillator

## Abstract

The wearable cardioverter-defibrillator (WCD), or LifeVest (ZOLL Medical Corporation, an Asahi Kasei company, Tokyo, Japan), is designed for continuous monitoring and immediate treatment of patients at high risk of sudden cardiac arrest (SCA). Due to a lack of water resistance and inability to operate safely in wet conditions, current guidelines recommend wearing the device at all times except for short periods, such as during showering. We present the case of an 88-year-old male with complex coronary artery disease, ischemic cardiomyopathy, and permanent atrial fibrillation who experienced cardiac arrest and ventricular fibrillation (V-Fib) during a shower when not wearing his LifeVest. Immediately after he lost consciousness, his wife promptly reattached the LifeVest, which stabilized his condition with a shock delivered by the device. This case highlights potential risks during device-free periods and the limited data on compliance and outcomes in these intervals. Further study is needed to assess risks and explore solutions that allow for the device's removal for short periods and enhance the patient’s safety.

## Introduction

The wearable cardioverter-defibrillator (WCD), commonly known as the LifeVest (ZOLL Medical Corporation, an Asahi Kasei company, Tokyo, Japan), provides continuous monitoring and immediate treatment for patients at high risk of sudden cardiac arrest (SCA) and serves as a bridge to ICD implantation or during early recovery when immediate placement is not feasible [1-3]. Current guidelines recommend that patients should wear the device continuously to ensure maximum protection against sudden cardiac arrest, but the device can be removed for short periods during activities such as showering or bathing [4]. Despite the recommended consistent use of the device, there have been cases witnessed where patients encountered unexpected adverse cardiac events during device-off periods. The VEST trial indicated that 64% of arrhythmic deaths in patients assigned to the WCD occurred when the device was not being worn [5]. 

We discuss a case involving a patient with a history of complex cardiovascular disease who experienced cardiac arrest and ventricular fibrillation (V-fib) during showering when not wearing his LifeVest. 

## Case presentation

An 88-year-old male with a history of coronary artery disease (CAD), systolic heart failure with the left ventricular ejection fraction (LVEF) of 15-20%, and permanent atrial fibrillation underwent multiple percutaneous coronary interventions (PCIs) with stent placements in the right coronary artery (RCA) and left anterior descending (LAD) artery. Due to his severe cardiac conditions and high risk of ventricular arrhythmias, he was prescribed a LifeVest, and he wore it continuously until he received an implantable cardioverter-defibrillator (ICD) two weeks later. 

**Figure 1:**
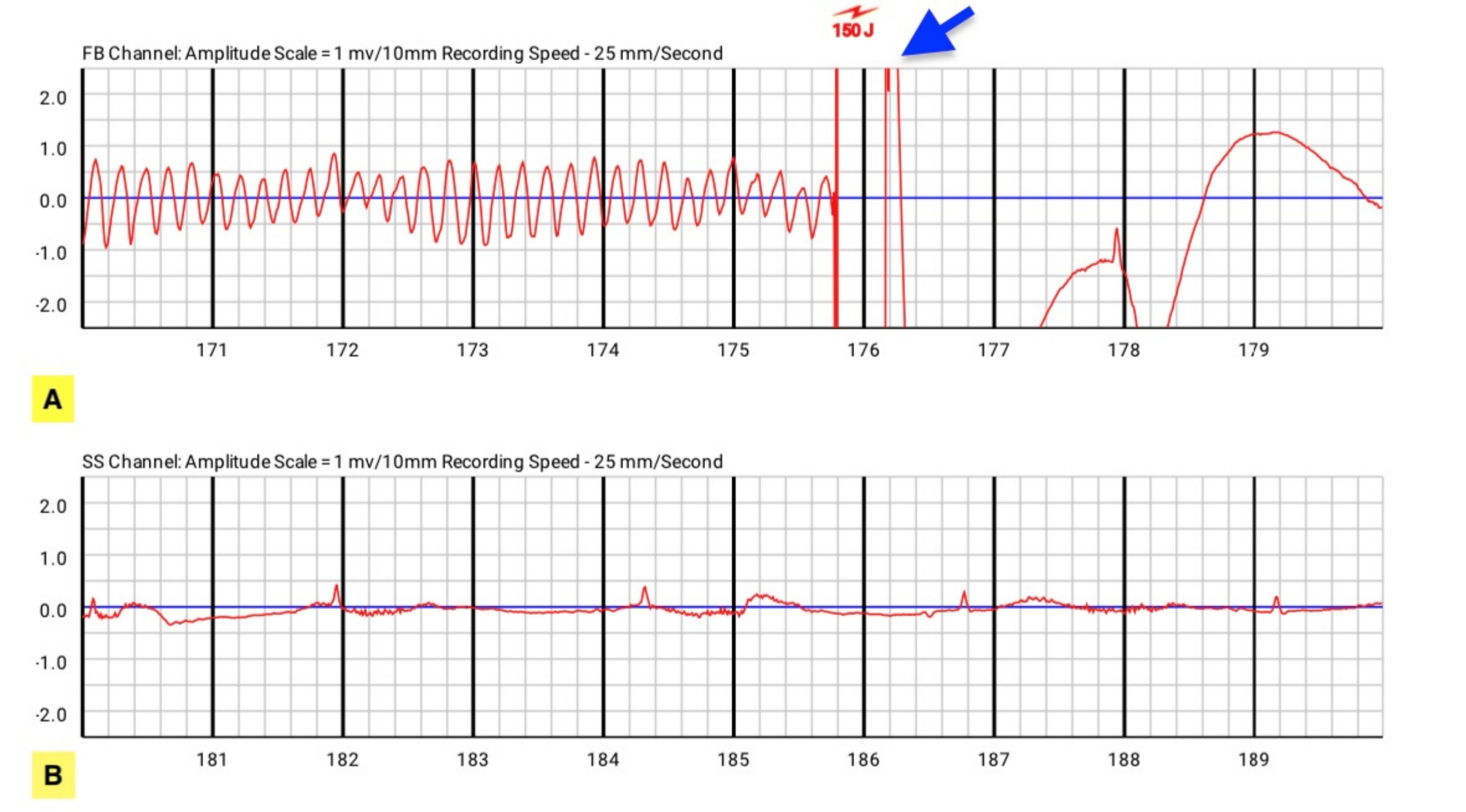
ECG strips showed that the patient had a V-fib event and was resuscitated with LifeVest. (A) A V-fib event was terminated by a LifeVest shock of 150 J (blue arrow), resulting in restoration of normal sinus rhythm. (B) Fifteen minutes after the event, normal sinus rhythm remains stable.

One week after initiating use of the device, he developed sudden cardiac arrest (SCA) and V-fib while his LifeVest was removed for showering. Immediately after he lost consciousness, his wife promptly reattached the LifeVest. After being reapplied, real-time electrocardiogram (ECG) monitoring resumed instantly; the device quickly recognized the active arrhythmia and stabilized his condition by delivering a shock. Twenty minutes later, the patient was transported to the emergency department for further evaluation and management. ECG strips showed that the patient had a V-fib event and was resuscitated with LifeVest (Figure 1).

During this hospital visit, due to his atrial fibrillation and subsequent cardiac events, he was started on amiodarone for immediate rhythm stabilization while awaiting definitive ICD implantation. Emergency Medical Services (EMS) had to shock him twice en route to the hospital and provide cardiopulmonary resuscitation (CPR). He was stabilized in the hospital and arranged for an ICD implantation.

After a four-day hospital stay, the patient received the ICD, which provided continuous protection against future cardiac events, eliminating the need for the LifeVest. He continued to be monitored closely. Records from the last follow-up said that he was overall well with no noticeable symptoms such as chest pain, shortness of breath, or edema. In addition to an adjustment in his medication regimen, he was also provided with education on the management of his conditions and the importance of compliance with his treatment plan.

## Discussion

Sudden cardiac arrest (SCA) caused by ventricular tachyarrhythmias is a major cause of mortality among heart disease patients, with approximately 356,000 deaths annually in the United States [6]. It is well established that implantable cardioverter defibrillators (ICDs) are life-saving devices that provide essential protection against SCA. The Multicenter Automatic Defibrillator Implantation Trial II (MADIT-II) found that prophylactic ICD implantation reduced mortality in patients with myocardial infarction and reduced ejection fraction [7]. However, situations like recent revascularization or cardiomyopathy may not allow immediate ICD implantation, hence leaving patients at risk of encountering SCA [8]. In such cases, the WCD, like LifeVest, is proven crucial since it provides temporary protection, an essential safety net that delivers immediate defibrillation shocks when life-threatening arrhythmias are detected [9,10]. Indeed, the WCD’s effectiveness in preventing sudden cardiac death among high-risk patients has been well-documented by the WEARIT-II Registry. Revelations of data from the registry indicate that 1.6% of the 2000 patients experienced appropriate WCD shocks, with a survival rate of 92% for those who received an appropriate shock. Through such findings, it is illustrated that the WCD serves as a crucial bridge during the period until patients can receive an ICD or achieve myocardial recovery [11].

With the benefits of the WCD, the FDA approved the device in 2002, acknowledging its potential to save lives in patients at risk of SCA [4]. This approval stemmed from the result of the FDA’s study, which involved 289 patients from the US and Europe wearing the device for an average of 20 hours daily over three months. These patients, who were awaiting heart transplants or had recently experienced heart attacks or undergone coronary bypass surgeries, demonstrated a 71% success rate in treating sudden cardiac arrest, specifically in the termination of life-threatening arrhythmia such as ventricular tachycardia or fibrillation. Current guidelines report that the wearable cardioverter defibrillator (WCD) should be worn 24 hours a day, except during bathing or showering. In addition, users needed to transfer data to their monitoring hospital weekly via modem.

However, despite the visible advantages of the WCD against potential cardiac arrests, it still remains a challenge to ask for compliance from patients regarding the consistent use of the device. One of the explanations for such non-compliance could be a decrease in life quality, seen by the presence of discomfort, skin irritation, and emotional distress, that wearing the WCD has caused [12,13]. Another possible explanation is the potential for adverse, and even fatal, outcomes resulting from the interaction between LifeVest and other cardiac devices, such as pacemakers. LaPage et al. reported a case study of a 17-year-old patient who experienced inappropriate shocks from the wearable defibrillator, which were triggered by the signals from the unipolar ventricular pacemaker. Despite medical intervention, the interaction led to a fatal outcome [14]. That said, compliance is a crucial factor in the effectiveness of the WCD. Patients typically wore the device for an average of 21.3 hours per day, reflecting generally high compliance rates [12]. However, there were still instances when the device was not worn, mainly due to discomfort or during certain activities. Even these brief periods of non-compliance can significantly elevate the risk of adverse events such as arrhythmias [9].

In the case of our patient, the 88-year-old male with a long history of complex coronary artery disease, ischemic cardiomyopathy, and permanent atrial fibrillation, encountered SCA and V-fib while his LifeVest was removed for showering. When witnessing the situation, his wife immediately reattached the LifeVest, whose shock managed to stabilize his condition until further medical assistance. The unexpected occurrence of V-fib catching the patient off guard in such a short period of time underlines the need to dedicate more attention to the necessity of the LifeVest being present at all times. Due to the limited data on compliance and outcomes in these intervals, further study is needed to assess risks and explore solutions that allow for the device's removal for short periods and enhance the patient's safety. Advancements such as waterproof coverings and continuous monitoring technology could enable safer use of LifeVest during showers, minimizing the time patients are without protection. Recent clinical studies have demonstrated that the water-resistant, patch-based wearable cardioverter-defibrillator (P-WCD) enables continuous daily use, including during showering, with high patient compliance and effective protection against SCA [5]. In addition, individualized risk assessments are crucial for identifying patients who may need stricter usage guidelines; for instance, those with a history of frequent arrhythmias or severely reduced ejection fractions might require more stringent protocols to minimize risk, as well as implementing shorter but more frequent monitoring intervals. The VFRisk score, recently validated in the Framingham Heart Study, integrates 12 clinical risk factors, including heart failure, atrial fibrillation, prior myocardial infarction, diabetes, and stroke, to stratify sudden cardiac death (SCD) risk with high accuracy (C-statistic 0.71). Notably, individuals in the highest risk quartile experienced nearly eightfold higher SCD odds than those at lowest risk, underscoring the value of this comprehensive, individualized approach for patients at risk and those using wearable defibrillators [15].

To further encourage patients to comply with consistent LifeVest use, education in the form of patient counseling and support groups could be made more prevalent. By dispersing information about how to alleviate emotional and physical discomfort when wearing the device, patients would be able to improve their experiences with LifeVest and hence be able to wear them consistently [16]. These strategies can improve patient compliance by ensuring that patients understand the critical role of LifeVest in preventing SCA. In addition to education that accelerates compliance of patients in utilizing LifeVest, education on how the device should be appropriately used is also required. Adler et al. found that up to 10% of patients who received inappropriate shocks forgot instructions, and others did not place electrodes properly after removal [17].

Furthermore, a valuable enhancement to the current model is the ability to activate an emergency response system to the nearest healthcare facility upon recognizing V-tach or V-fib and delivering a shock. This could reduce poor outcomes after ventricular arrhythmias, as patients may be unable to call for help [16]. Besides, more comfortable and user-friendly LifeVest designs could also be implemented to improve the experiences of the patient with the device.

## Conclusions

This case illustrates an important gap in our understanding of the clinical safety of short-term LifeVest removal, particularly during activities such as showering. Although temporary removal is permitted under current recommendations, the risk of life-threatening arrhythmias during these brief, unmonitored intervals remains insufficiently characterized. Continuous monitoring, patient education, and possibly technological advancements in LifeVest design, such as P-WCD, are essential steps toward minimizing risks for high-risk cardiac patients. Further studies, particularly large-scale observational registries and device performance studies, are needed to evaluate the impact of continuous LifeVest usage on patient outcomes and to develop more comprehensive guidelines.
